# Xanthine and 8-oxoguanine in G-quadruplexes: formation of a G·G·X·O tetrad

**DOI:** 10.1093/nar/gkv826

**Published:** 2015-09-22

**Authors:** Vee Vee Cheong, Brahim Heddi, Christopher Jacques Lech, Anh Tuân Phan

**Affiliations:** School of Physical and Mathematical Sciences, Nanyang Technological University, 637371 Singapore

## Abstract

G-quadruplexes are four-stranded structures built from stacked G-tetrads (G·G·G·G), which are planar cyclical assemblies of four guanine bases interacting through Hoogsteen hydrogen bonds. A G-quadruplex containing a single guanine analog substitution, such as 8-oxoguanine (O) or xanthine (X), would suffer from a loss of a Hoogsteen hydrogen bond within a G-tetrad and/or potential steric hindrance. We show that a proper arrangement of O and X bases can reestablish the hydrogen-bond pattern within a G·G·X·O tetrad. Rational incorporation of G·G·X·O tetrads in a (3+1) G-quadruplex demonstrated a similar folding topology and thermal stability to that of the unmodified G-quadruplex. pH titration conducted on X·O-modified G-quadruplexes indicated a protonation-deprotonation equilibrium of X with a pKa ∼6.7. The solution structure of a G-quadruplex containing a G·G·X·O tetrad was determined, displaying the same folding topology in both the protonated and deprotonated states. A G-quadruplex containing a deprotonated X·O pair was shown to exhibit a more electronegative groove compared to that of the unmodified one. These differences are likely to manifest in the electronic properties of G-quadruplexes and may have important implications for drug targeting and DNA-protein interactions.

## INTRODUCTION

The human genome has a high occurrence of G-rich sequences, notably in oncogenic promoters, mini-satellites and telomeric regions (1–6). For example, the human telomeres comprise of thousands of d(GGGTTA) repeats (7), reaching a total length of 3–20 kilobases (8). *In vitro*, these G-rich sequences can fold into stable four-stranded DNA structures called G-quadruplexes (9,10) with various folding topologies (6,7). Recently, the visualization of G-quadruplexes in human cells using G-quadruplex-specific antibodies was reported (11,12).

**Figure 1.**
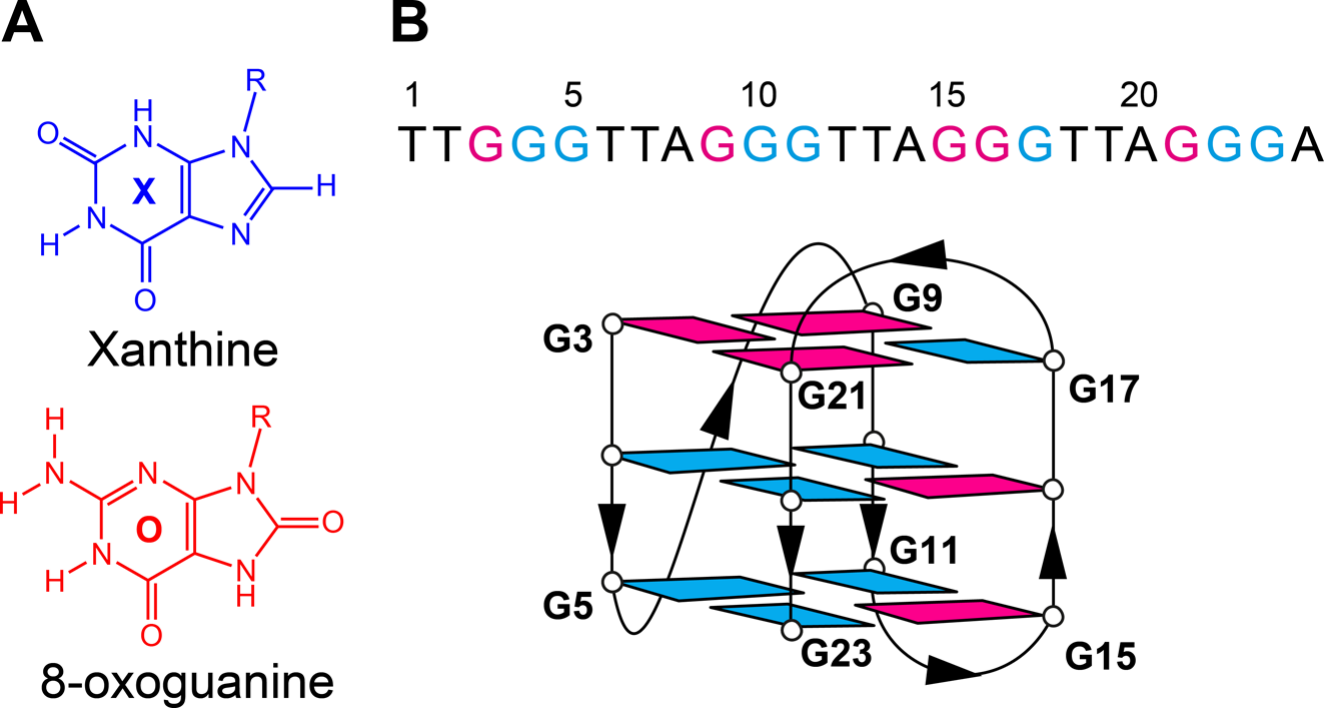
(**A**) Chemical structure of guanine analogs xanthine (X) and 8-oxoguanine (O). The R group in the chemical drawings refers to 2′-deoxyribose. (**B**) Top: sequence of *HT* with its base numbering shown above. Bottom: schematic structure of *HT*. *Syn* and *anti* guanines are colored in magenta and cyan, respectively.

Numerous studies have investigated the incorporation of guanine base analogs into G-quadruplexes (13–38). This study deals with two major naturally occurring guanine base lesions: 8-oxoguanine and xanthine. The lesion 8-oxoguanine (abbreviated as O, Figure 1A) is formed in cells via oxidative damage of the guanine base (39,40). Single G-to-O substitutions were reported to destabilize G-quadruplexes (30,35,37,41,42). In contrast, little is known about the effects of xanthine, another major guanine analog formed via nitrosative deamination of a guanine base (abbreviated as X, Figure 1A) (43,44), when incorporated within G-quadruplexes.

**Figure 2.**
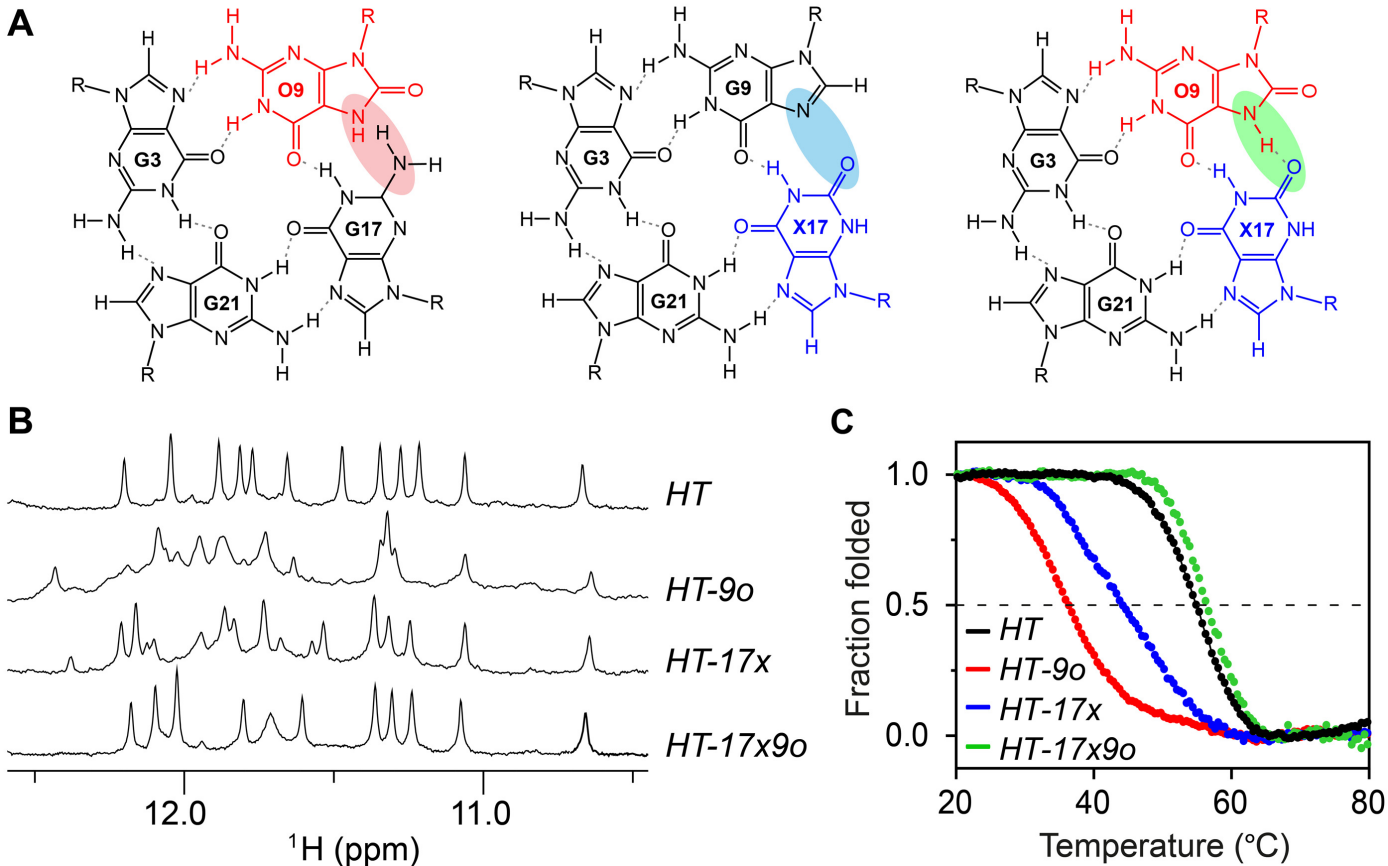
Characterization of *HT* and the modified sequences *HT-9o*, *HT-17x* and *HT-17x9o*. (**A**) Arrangement of the top G-tetrad with modification O at position 9 (left), X at position 17 (middle), and both O and X at position 9 and 17 respectively (right). Hydrogen bonds are represented by dotted lines. Hydrogen-bond connectivity affected by the modification(s) is shaded. (**B**) Imino proton spectra and (**C**) UV melting curves of these sequences.

In theory, a point mutation of either O or X within a G-tetrad would result in a loss of a hydrogen bond and/or potential steric hindrance (Figure 2A). However, the disruptive effects of a single modification can be alleviated by concurrent X and O substitutions in a proper orientation where the hydrogen donor/acceptor pattern at the Hoogsteen face of O is complementary with that of the Watson-Crick face of X (Figure 2A). The formation of a G-quadruplex containing an X·O·X·O tetrad was suggested by Hartig and co-workers (29,45). However, the formation of such a modified tetrad has not yet been confirmed by a high-resolution structural study. Furthermore, the thermal stability and circular dichroism data of these modified G-quadruplexes indicated the formation of less stable G-quadruplex structures or multiple conformations, possibly linked to the fact that an X·O·X·O tetrad often constrained O in an unfavorable *anti* orientation (46,47).

In this study, we explore the effects of O and X mutations to a modified human telomeric sequence, termed *HT* (Table 1), which adopts a well-defined (3+1) G-quadruplex (48). Using NMR spectroscopy, we determined the solution structure of a G-quadruplex containing a G·G·X·O tetrad and affirmed the complementary arrangement between X and O to recover the hydrogen-bond loss which would have occurred with a single guanine analog. We show that O can be rationally incorporated in a *syn* position to achieve high G-quadruplex thermal stability. Our NMR data revealed the X base in a X·O-modified G-quadruplex to exist in both protonated and deprotonated states at physiological pH. A consequence of the deprotonation of X is a negative charge manifested within a groove, which has potential implications for drug binding and DNA-protein interactions.

**Table 1.** Dual-substituted G-quadruplex sequences and their thermal stability

Name	Sequence (5′-3′)				*T_m_* (°C)	Δ*T_m_* (°C)
	*SAA*	*SAA*	*SSA*	*SAA*		
*HT*	TT GGG	TTA GGG	TTA GGG	TTA GGG A	55.6 ± 0.5	
*HT-3x21o*	TT **X**GG	TTA GGG	TTA GGG	TTA **O**GG A	50.8 ± 0.7	−4.8
*HT-9x3o*	TT **O**GG	TTA **X**GG	TTA GGG	TTA GGG A	52.2 ± 0.2	−3.4
*HT-17x9o*	TT GGG	TTA **O**GG	TTA GG**X**	TTA GGG A	57.4 ± 1.1	+1.8
*HT-10x16o*	TT GGG	TTA G**X**G	TTA G**O**G	TTA GGG A	55.9 ± 0.8	+0.3
*HT-11x15o*	TT GGG	TTA GG**X**	TTA **O**GG	TTA GGG A	55.3 ± 1.3	−0.3
HT-21x17o*	TT GGG	TTA GGG	TTA GG**O**	TTA **X**GG A	43.4 ± 1.2	−12.2

*T_m_* values were determined in buffer containing 10 mM potassium phosphate (pH 7.0) and 10 mM potassium chloride. The modified residues used are: X = xanthine; O = 8-oxoguanine.

The glycosidic conformation of each residue of the G-tetrad core is in *syn* (*S*) or *anti* (*A*) orientation.

*O was substituted at the disfavored *anti* glycosidic position in this construct.

## MATERIALS AND METHODS

### Sample preparation

Unlabeled and 4% site-specific ^15^N-labeled oligonucleotides were synthesized on an ABI 394 DNA/RNA synthesizer. Standard DNA phosphoramidites, solid supports and additional reagents were purchased from Glen Research Corporation. ^15^N-labeled phosphoramidites were purchased from Cambridge Isotope Laboratories. Cleavage from the solid support, deprotection and purification (by PolyPak II cartridges, Glen Research) of oligonucleotides were performed according to the manufacturer's protocols. Purified oligonucleotides were subsequently dialyzed successively in deionized water, 25 mM KCl, and again in deionized water, and freeze-dried. Oligonucleotides were dissolved in buffer comprising 20 mM potassium phosphate (KPi) (pH 5.0–9.0), 70 mM KCl and 10% D_2_O. DNA concentration was expressed in strand molarity. Extinction coefficient of the modified sequences was approximated to that of the unmodified sequence.

### Ultraviolet (UV) absorption spectroscopy

UV melting experiments of oligonucleotides were performed on JASCO V-650 spectrophotometer using a 1 cm pathlength quartz cuvette, monitoring the UV absorbance at 295 nm over a temperature range of 15–90ºC. Samples were prepared in 450 μl reaction volume, with 5 μM DNA in buffer containing 10 mM KPi (pH 7.0) and 10 mM KCl. This ionic condition was chosen in order to relate the measurements with those from the previous thermal stability studies of the same scaffold (21,35). The heating and cooling rates were set at 0.2ºC/min. All samples were heated to 90ºC for 15 min prior to the start of the experiments. All spectra were normalized by two baselines corresponding to the completely folded (low temperature) and fully unfolded (high temperature) state. The melting temperature (*T_m_*) was defined as the temperature, at which there were equal populations of the folded and unfolded species. *T_m_* values were averaged from the heating and cooling curves. Hysteresis of less than 3.0°C was observed for all samples.

### Circular dichroism (CD) spectroscopy

CD spectra were recorded with 5 μM DNA samples in buffer containing 10 mM KPi (pH 7.0) and 10 mM KCl, on a JASCO-815 spectropolarimeter at 20ºC over the wavelength range of 220–320 nm using a 1 cm pathlength quartz cuvette. Each spectrum was averaged from an accumulation of three scans, baseline corrected, and zero-corrected at 320 nm.

### Nuclear magnetic resonance (NMR) spectroscopy

NMR experiments were performed on 600-MHz and 700-MHz Bruker AVANCE spectrometers at 25ºC, unless stated otherwise. Samples were prepared in buffer containing 20 mM KPi (pH5.0–9.0), 70 mM KCl and 10% D_2_O, with DNA strand concentration typically ranging from 0.1 to 0.7 mM. NMR spectra of *HT* at a lower salt condition (10 mM KPi and 10 mM KCl) were also recorded and no significant changes were observed. The chemical shifts were calibrated against DSS (4,4-dimethyl-4-silapentane-1-sulfonic acid). Imino protons (H1) of guanines were unambiguously assigned via site-specific low enrichment (49), and through-bond correlation at natural abundance (50,51). Spectral assignments were assisted by NOESY, TOCSY, COSY and {^1^H-^13^C}-HSQC experiments. All spectra were processed by Topspin 2.1 (Bruker) and analyzed by SPARKY 3.115 (52).

### NMR pH titration

Samples used in pH titration experiments were prepared at DNA concentration of 0.15–0.2 mM with the required pH adjusted using HCl and/or LiOH and mixed thoroughly before a 1D NMR spectrum was recorded as described above. The NMR pH titration curves were fitted with a sigmoidal function.

### Computation of NMR chemical shifts

Computed chemical shifts of G-tetrad systems were obtained using the Gaussian 03 Software (53). Model G·G·X·O and G·G·X(-)·O nucleobase tetrads containing two centrally located K^+^ ions were minimized at the HF/6-31G(d) and MP2/6-31G(d) levels (54). NMR chemical shifts were then computed for optimized tetrads using the GIAO method (55) at the HF/6-31G(d) level and calibrated using TMS as a reference.

### Structure calculation

NMR-constrained structure calculations of DNA G-quadruplexes were performed with the program XPLOR-NIH (56) in two general steps: (i) distance geometry simulated annealing and (ii) restrained molecular dynamics refinement. Constraints imposed on the simulation include hydrogen-bond restraints, inter-proton distance restraints, dihedral restraints, planarity restraints and repulsive restraints. Inter-proton distances were deduced from NOESY experiments performed in H_2_O (mixing time, 300 ms) and in D_2_O (mixing time, 100 ms and 300 ms). The 10 lowest-energy structures were selected and displayed using the program PYMOL (57).

### Molecular dynamics (MD) refinement

Lowest-energy structures from the XPLOR-NIH calculations underwent further MD refinement in explicit solvent using the Amber 10 software (58). Systems were neutralized by the introduction of 23 K^+^ ions and solvated using TIP3P water molecules (59) in a truncated octahedral box. Systems underwent an initial 500 steps of steepest decent minimization followed by 500 steps of conjugated gradient minimization with harmonic potential position restraints of 25 kcal.mol^−1^.Å^−2^ on solute atoms. Systems were then heated from 100 to 300 K under constant volume over 10 ps while maintaining restraints. Subsequent minimization and equilibration cycles were performed, in which position restraints on solute atoms were gradually reduced to 5, 4, 3, 2, 1 and 0.5 kcal.mol^−1^.Å^−2^. Final simulations were run in the absence of position restraints at 300 K and 1 atm for 1 ns. Intra-proton distance restraints based on NMR restraints were imposed throughout these simulations. DNA coordinates were extracted from MD simulations and briefly minimized in vacuum. The 10 lowest-energy structures were chosen for final presentation. Partial atomic charges used in MD refinement for non-native DNA residues 8-oxoguanine (O) and xanthine (X) were calculated using the RESP procedure at the HF/6-31G(d) level through the RED software (60).

### Poisson-Boltzmann calculations

Electrostatic surface potentials of *HT-17x9o* and *HT* G-quadruplexes were calculated using the APBS tool (61) of PYMOL (57), through the linearized Poisson-Boltzmann equation. An ionic strength of 0.15 M, temperature of 37°C, and solvent dielectric constant of 78.0 were used as parameters. Atomic partial charges used in Poisson-Boltzmann equation were taken from those generated using Amber.

### Data deposition

The coordinates of the G-quadruplex structure formed by the *HT-17x9o* sequence d[TTGGGTTAOGGTTAGGXTTAGGGA] at pH 8.0 have been deposited in the Protein Data Bank (accession code 2MWZ).

## RESULTS

### Single substitutions of guanine (G) with 8-oxoguanine (O) and xanthine (X) are highly disruptive to G-quadruplexes

We performed single O substitutions at positions G9, G16 and G17 of the (3+1) G-quadruplex scaffold formed by the *HT* sequence (Figure 1 and Table 1). The number and intensities of peaks in the imino proton NMR spectra of these sequences (Figure 2B and Supplementary Figure S1) indicated the co-existence of multiple G-quadruplex forms. In agreement with the NMR data, their CD spectra show notable deviation from that of the unmodified *HT* sequence (Supplementary Figure S1). The melting temperature (*T_m_*) of these O-substituted sequences is greatly reduced (19–31°C) as compared to that of *HT* (Figure 2C and Supplementary Table S1), supporting the previous finding that O substitutions highly destabilize G-quadruplex structures. This destabilization effect may come from a steric clash between the proton H7 of 8-oxoguanine and the amino group of the neighboring guanine (Figure 2A).

Although previous computational studies suggested that tetrads formed entirely by xanthine (X) are possible (62,63), no experimental studies on the effects of single X substitutions in G-quadruplexes have been reported. Here we systematically performed single X substitutions at all twelve G positions of the *HT* sequence. NMR and CD spectra of X-substituted sequences (Figure 2B and Supplementary Figure S2) indicate that a G-to-X modification induces the formation of multiple G-quadruplexes in most cases (except for substitutions at G10 and G16). Melting experiments show that single X substitutions also highly destabilize G-quadruplex structures, exhibiting a *T_m_* decrease of over 10°C as compared to that of *HT* (Figure 2C and Supplementary Table S1). This destabilizing effect is most likely due to a hydrogen-bond loss and unfavorable electrostatic interaction in the context of a single X substitution (Figure 2A). As a comparison, a milder destabilizing effect was observed for inosine (I) substitutions, which appeared to maintain the same G-quadruplex fold in most cases and caused smaller decreases in the melting temperature (Supplementary Table S1 and Figure S3).

### Complementary X·O dual substitutions are compatible with G-quadruplexes

While individual single substitutions of O at position G9 and X at position G17 (the sequences *HT-9o* and *HT-17x*, respectively; Supplementary Table S1), were highly disruptive to the G-quadruplex structure, simultaneous substitutions of O and X at these positions, resulting in the sequence *HT-17x9o* (Table 1), allowed for the formation of a G·G·X·O tetrad, which restored the hydrogen bonding integrity of the G-tetrad via complementary pairing between X and O. In this tetrad, the X·O pair is linked by two hydrogen bonds: one formed between N1-H1 of xanthine and the C=O group of 8-oxoguanine; the other formed between N7-H7 of 8-oxoguanine and the C=O group of xanthine (Figure 2A).

The imino proton NMR spectrum of *HT-17x9o* shows a well-resolved spectrum with 10 sharp peaks (and a broad peak), indicating the formation of a single G-quadruplex conformation (see further analysis below). The NMR chemical shift pattern (Figure 2B) and CD spectrum (Supplementary Figure S4) of *HT-17x9o* are highly similar to that of *HT*, consistent with the conservation of the (3+1) G-quadruplex folding topology. In sharp contrast to the strong destabilization observed for sequences with a single substitution *HT-9o* and *HT-17x*, the complementary X·O dual substitutions in *HT-17x9o* led to a recovery of the thermal stability of the G-quadruplex structure (Figure 2C), displaying a melting temperature 1.8°C higher than that of the unmodified *HT* sequence (Table 1).

As 8-oxoguanine was previously shown to favor a *syn* conformation (64), we generated four other X·O-modified sequences in which O were at the positions to adopt a *syn* conformation in the original structure (G3, G15, G16 and G21) (Table 1). All these X·O-dual substituted sequences (i.e., *HT-9x3o*, *HT-11x15o*, *HT-10x16o* and *HT-3x21o*) were found to exhibit similar characteristics as that of *HT-17x9o*, including the NMR spectra, CD spectra, and melting temperature, indicating that the same G-quadruplex form had been adopted (Supplementary Figure S4 and Table 1).

The sequence *HT-21x17o* was synthesized to probe the effect of substituting O into an *anti* position G17 (Table 1 and Supplementary Figure S5). The NMR and CD spectra of *HT-21x17o* were similar to those of *HT*, indicating that this sequence adopted predominantly the same (3+1) G-quadruplex fold despite a minor population being evident in NMR imino proton spectra (Supplementary Figure S5A). However, the melting temperature of *HT-21x17o* with O in an *anti* conformation at position 17 was significantly reduced (−12.2°C) compared to that of *HT-17x9o* with O substituted into a *syn* position 9 (Table 1), suggesting that O substitution within a G·G·X·O tetrad exhibits a preference for the *syn* conformation.

### Protonation states of a G·G·X·O tetrad

In the course of this study, we observed interesting changes in the imino proton NMR spectra as a function of pH, allowing us to probe the protonation states of the G·G·X·O tetrad. At pH 7.0, imino proton spectra of X·O dual substituted G-quadruplexes (Table 1) consistently exhibited only 10 major sharp peaks (Supplementary Figures S4 and S5) out of the 13 expected peaks in this region (12 H1 protons of G/X/O and the H7 proton of O involved in base pairing hydrogen bonds, Figure 2A). At pH 5.0 and pH 9.0, all 13 sharp peaks could be observed in this region (Supplementary Figure S6). As a comparison, the imino proton spectrum of the unmodified *HT* sequence showed 12 peaks for the entire range of pH from 5.0 to 9.0 (Supplementary Figure S6).

**Figure 3.**
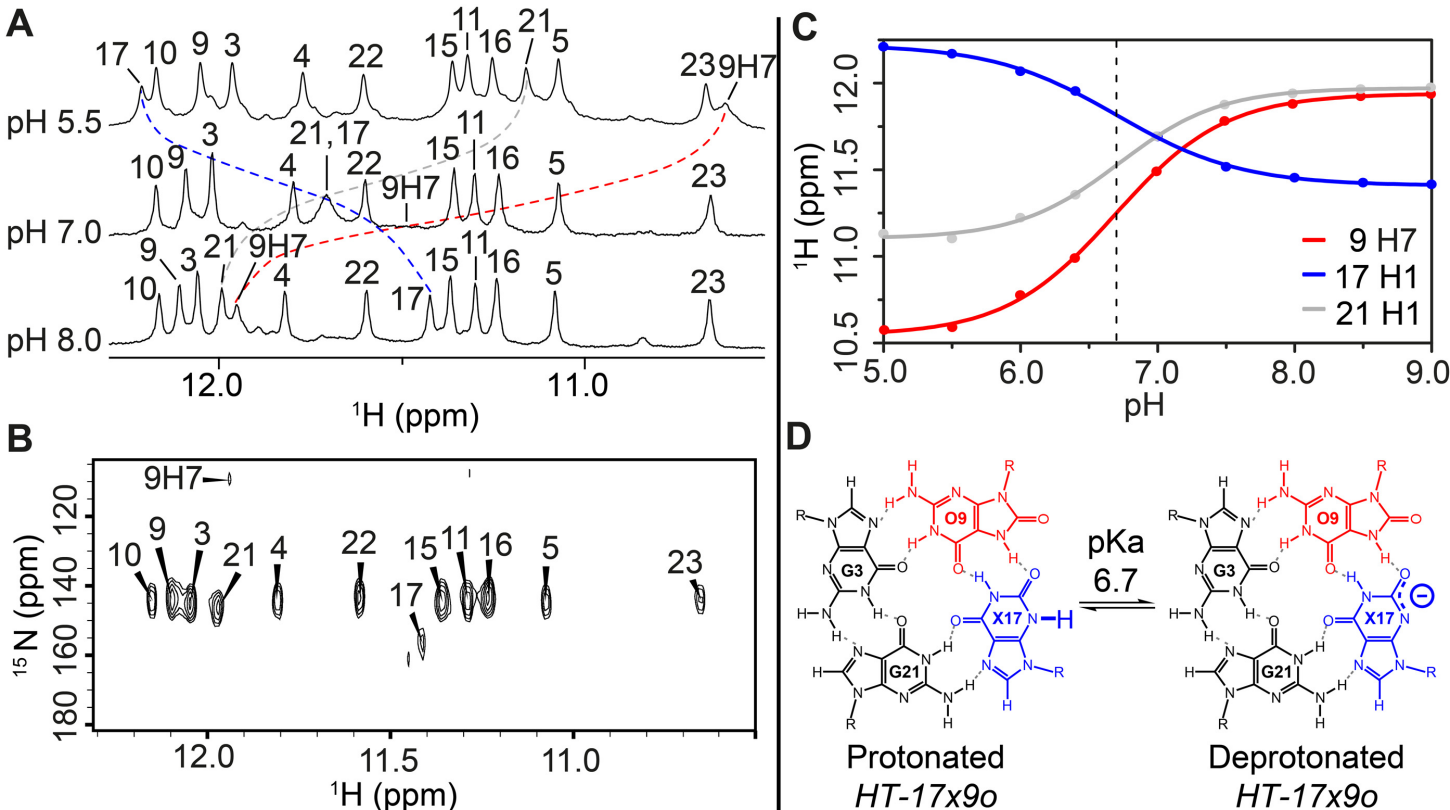
NMR pH titration of *HT-17x9o*. (**A**) Imino proton spectra of *HT-17x9o* at pH 5.5, 7.0 and 8.0. Identities of imino protons are indicated above each spectrum. Peak of O9H7 at pH 7.0 was broadened and not seen on the spectrum. (**B**) {^1^H-^15^N}-HSQC spectrum showing N1-H1 cross-peaks, which are labeled with their corresponding nucleotide number for *HT-17x9o* at pH 8.0. The N7-H7 cross-peak of O9 was also observed. (**C**) Plot of NMR chemical shifts observed for the O9H7, X17H1 and G21H1 protons of *HT-17x9o* as a function of pH in the range of 5.0–9.0, with the mid-transition point represented by a dashed line. Peak shifts of O9H7, X17H1 and G21H1 are colored in red, blue and grey, respectively. (**D**) Ionization of xanthine (X17) in *HT-17x9o*, with the neutral form (left) in equilibrium with the anionic form (right) at pH 6.7, corresponding to its acid dissociation constant (pKa).

We focused our study on the *HT-17x9o* sequence at different pH. NMR imino proton spectra recorded at pH 5.0–9.0 showed large chemical shift perturbations, which were not observed for the *HT* G-quadruplex (Figure 3A and Supplementary Figure S6). Spectral assignment and subsequent analysis of *HT-17x9o* revealed that three peaks (O9H7, X17H1 and G21H1) experienced large chemical shift changes upon the pH titration. The {^1^H-^15^N}-HSQC spectrum of *HT-17x9o* was used to verify the identity of O9H7 and X17H1 (Figure 3B). (Further discussions on the identification of these peaks are presented below.) A plot of chemical shifts of these protons as a function of pH gave a similar transition point of pH ∼6.7 for each proton (Figure 3C), with protons O9H7 and G21H1 shifted downfield and X17H1 shifted upfield. These observations are consistent with the protonation/deprotonation at position N3 of X17 (Figure 3D) and are supported by quantum chemical calculations of NMR chemical shifts for idealized geometries of a G·G·X·O tetrad in protonated/deprotonated form (Supplementary Figure S7).

In literature, the protonation states of X in the context of a G-quadruplex structure have remained unexplored, although the protonation behavior of isolated X nucleobases and nucleosides have been studied (Supplementary Figure S8), with pKa of N3 being ∼7.7 and 5.7, respectively (65,66). From our experimental data, we have determined the pKa of X17N3 in the *HT-17x9o* G-quadruplex to be ∼6.7 via the midpoint transition of NMR chemical shifts (Figure 3C), within the previously determined pKa range of 5.7–7.7 for xanthine nucleobase and nucleoside.

### *HT-17x9o* maintains the (3+1) G-quadruplex fold in both the protonated and deprotonated states

**Figure 4.**
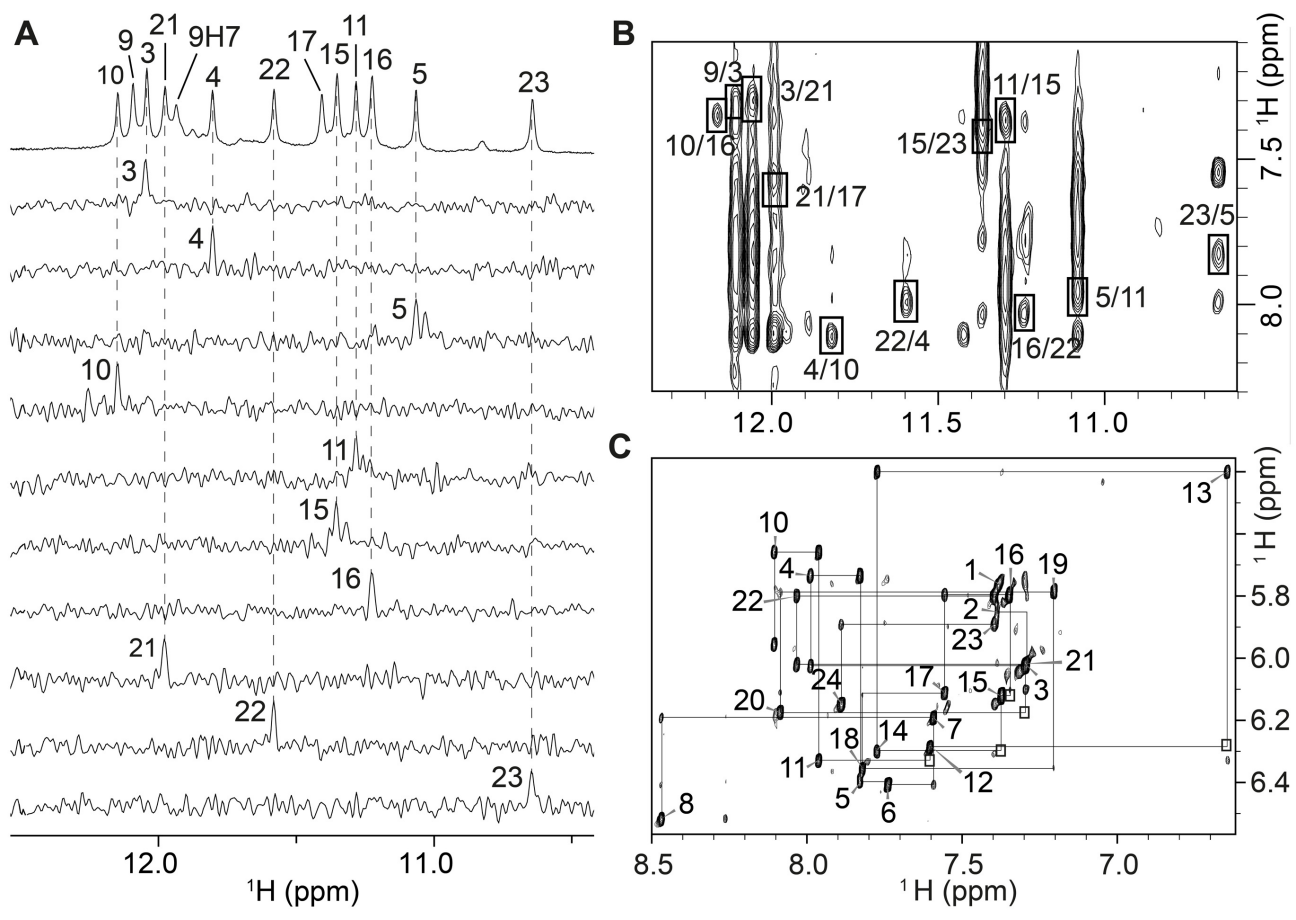
NMR spectra and assignments of *HT-17x9o* at pH 8.0. (**A**) Imino proton assignments based on ^15^N-filtered spectra recorded on samples with 4%-^15^N-enrichment at indicated positions. (**B**) NOESY spectrum of *HT-17x9o* (mixing time, 300 ms) used for folding topology determination. H1-H8 cross-peaks are framed and labeled with the residue number of imino protons followed by that of H8. (**C**) Sequential 5′-3′ walk on NOESY spectrum of *HT-17x9o*, based on H8/6-H1′ NOE traced by black lines. Intra-residue NOEs are labeled according to the respective nucleotide number. Connectivities not seen at this spectral threshold are framed.

We investigated the structural effects of incorporating a G·G·X·O tetrad into a G-quadruplex by determining the NMR solution structure of the *HT-17x9o* sequence. Guanines were 4% ^15^N-labeled (Supplementary Table S2) using a low-enrichment site-specific labeling approach (49). Guanine imino (H1) protons of *HT-17x9o* were unambiguously assigned at pH 5.5 (Supplementary Figure S9A), pH 7.0 (Supplementary Figure S10A) and pH 8.0 (Figure 4A). Assignment of the H8/6 protons were carried out at pH 7.0 (Supplementary Figure S10A) and extrapolated for pH 5.5 and 8.0, as no significant chemical shift changes for these protons were observed as a function of pH. Resonance assignments of O9 and X17 were assisted by NOESY (Supplementary Figure S11) and {^1^H-^15^N}-HSQC experiments at pH 8.0, where chemical shifts for O9H7 and X17H1 are distinct from those of guanines (Figure 3B). Aromatic and sugar protons were assigned in H8/H6-H1′ NOE sequential connectivity of *HT-17x9o*, and assisted by through-bond experiments (TOCSY, COSY and {^1^H,^13^C}-HSQC; data not shown) and through-space experiments (NOESY; Figure 4C). The adoption of *syn* glycosidic orientation by G3, G15, G16 and G21 were verified based on the high intensity observed for intra-residue H8-H1′ NOE cross-peaks (Figure 4C). The remaining guanines and xanthine X17 adopt the *anti* glycosidic conformation.

The H1 and H8 assignments of guanine, xanthine and 8-oxoguanine bases in *HT-17x9o* were applied to determine the alignment of three G-tetrads. At pH 8.0, the characteristic H1-H8 cyclic NOE connectivity pattern recorded for *HT-17x9o* (Figure 4B) could be identified for three G-tetrads: top, G3·G21·X17·O9; middle, G4·G10·G16·G22; and bottom, G5·G11·G15·G23. Imino protons from the G4·G10·G16·G22 tetrad were most protected from the exchange with solvent (data not shown), thus verifying their positioning as the middle G-tetrad. The hydrogen-bond directionality of the top G-tetrad (except for the hydrogen bond formed between O9H7 and X17O2) is opposite that of the other two G-tetrads, with the glycosidic conformations of residues from the top G-tetrad being *syn*·*syn*·*anti*·*syn*, and those of the middle and bottom G-tetrad being *anti*·*anti*·*syn*·*anti*. The G3–G5, O9–G11 and G21–G23 tracts are oriented in the same direction, while the G15–X17 tract is oriented in the opposite direction. The three loops (T6–A8, T12–A14 and T18–A20) are successively propeller, edgewise and edgewise, respectively.

Overall, the G-quadruplex folding topology of *HT-17x9o* is identical to that of *HT* (48), indicating that the replacement of two guanines with an X·O pair did not disturb the original (3+1) G-quadruplex scaffold. Furthermore, the H1-H8 NOE patterns recorded at pH 5.5 (Supplementary Figure S9B) and pH 7.0 (Supplementary Figure S10B) are similar to those observed at pH 8.0 (Figure 4B), indicating that both the protonated and deprotonated forms of *HT-17x9o* conserve the (3+1) G-quadruplex folding topology of the unmodified sequence (Supplementary Figures S9C and S10C).

### Solution structure of *HT-17x9o*: maintaining the shape and varying the groove charge

**Figure 5.**
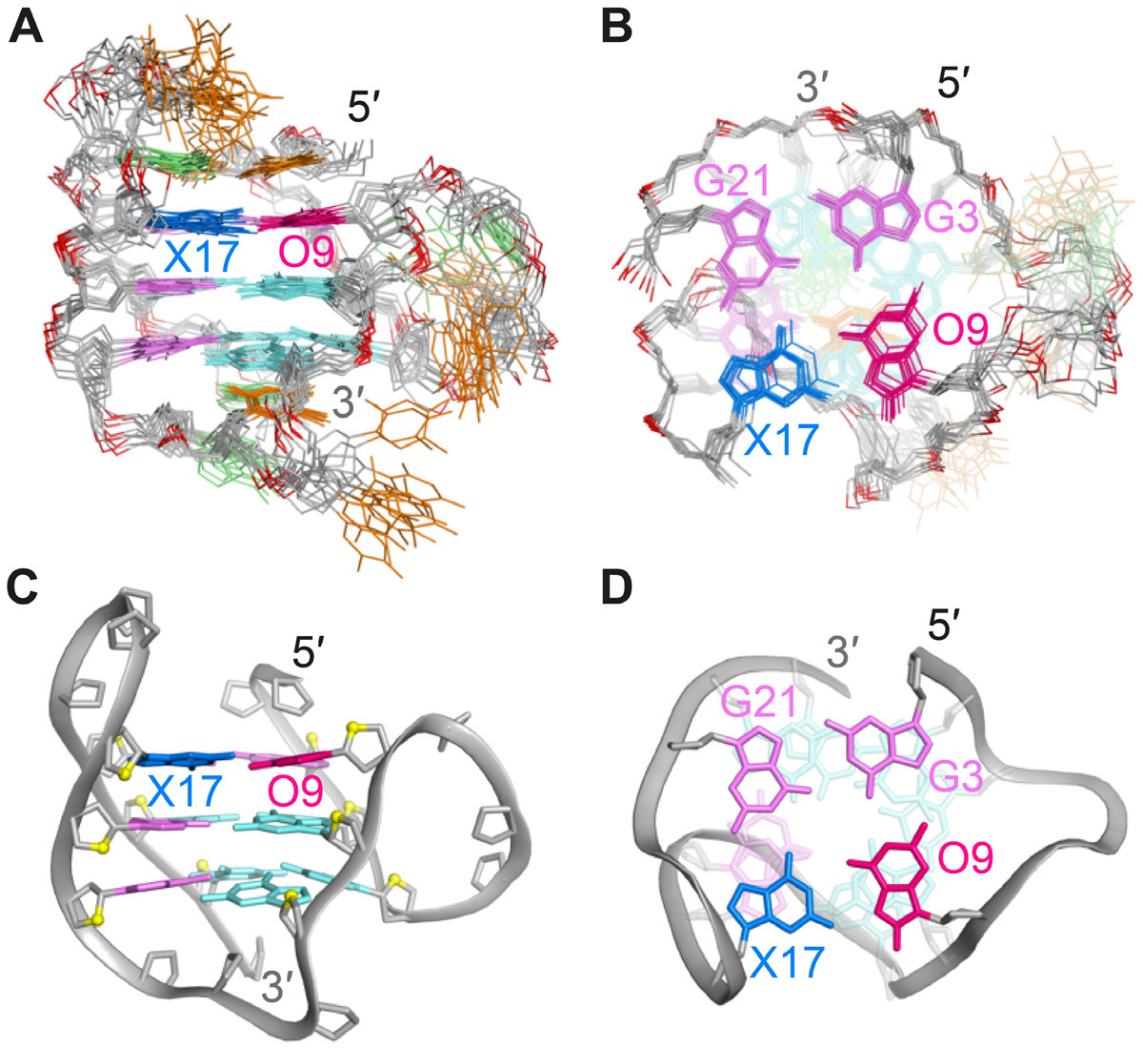
(**A**) Side view and (**B**) top view of 10 superimposed lowest energy conformations of *HT-17x9o*. Adenines are colored in green; thymines, orange; backbone and sugars, grey; O4′ atoms, yellow and phosphorus atoms, dark red. O9 and X17 are colored in red and blue, respectively. *Anti* and *syn* guanines are colored in cyan and magenta. (**C**) Side view and (**D**) top view of a representative structure of *HT-17x9o*. Residues 1, 2, 18 and 19 are removed in the top view for clarity.

We proceeded to solve the structure of the deprotonated form (at pH 8.0) of *HT-17x9o*. The solution structure of *HT-17x9o* (Figure 5) was computed on the basis of NMR constraints (Table 2 and Figure 4). The G-tetrad core consists of one narrow, one wide and two medium grooves, with the first TTA linker (propeller loop) traversing across a medium groove, while the T12–A14 and T18–A20 edgewise loops cross a wide groove at the bottom and a narrow groove at the top, respectively. Hydrogen-bond interaction between the 5′ and 3′ flanking nucleotides and the two edgewise loops is observed in a Watson-Crick T1·A20 and a reverse Watson-Crick T13·A24 base pair, with the former and the latter capping the top and the bottom of the G-tetrad core, respectively. These structural features are similar to those observed in the original *HT* G-quadruplex (Supplementary Figure S12).

**Table 2.** Statistics of the computed structures of the X·O-modified human telomeric sequence *HT-17x9o*

**A. NMR restraints**			
Distance restraints	D_2_O		H_2_O
Intraresidue	279		1
Sequential (*i*,*i* + 1)	153		11
Long-range (*i*, ≥*i* + 2)	40		43
Other restraints			
Hydrogen bond		64	
Dihedral angle		12	
**B. Structure statistics**			
NOE violations			
Number (>0.2 Å)		0.400±0.490	
Maximum violation (Å)		0.174±0.018	
RMSD of violations (Å)		0.023±0.002	
Deviations from the ideal covalent geometry			
Bond lengths (Å)		0.004±0.000	
Bond angles (°)		0.773±0.018	
Impropers (°)		0.418±0.002	
Pairwise all heavy atom RMSD values (Å)			
All heavy atoms except T and A residues		0.850±0.140	
All heavy atoms		1.660±0.320	

**Figure 6.**
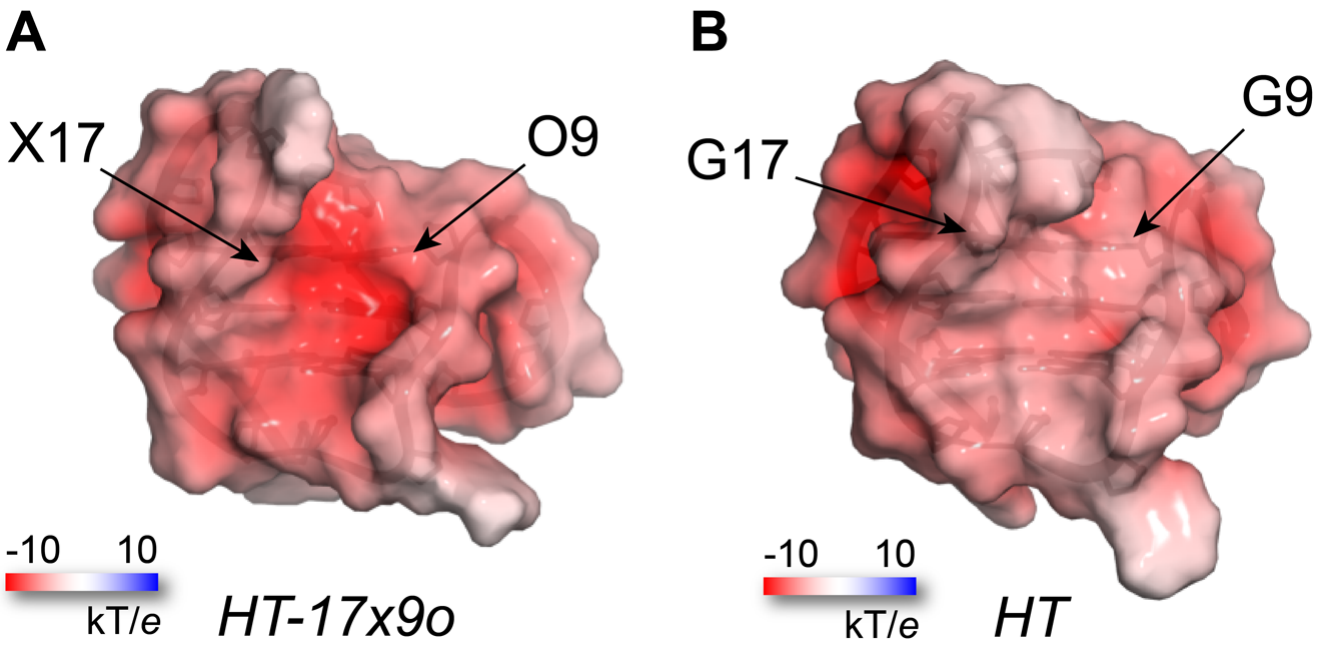
Electrostatic potential surface representation of (**A**) *HT-17x9o* and (**B**) *HT* G-quadruplexes, showing charge differences in the wide groove. Surface representation on both G-quadruplexes is presented at half transparency, whereby electrostatic potentials are displayed at ±10 kT/*e*, with white and red color indicative of neutral and negative charge potential, respectively.

However, a particular feature of introducing an X·O pair into a G-quadruplex is the ability to modulate a deprotonated and negatively charged xanthine base as a fucntion of pH. We computed the partial charge of X and O nucleotides and calculated the electrostatic potential at the surface of G-quadruplexes (see Materials and Methods). Comparative electrostatic potential surface representations between the *HT-17x9o* and the *HT* G-quadruplexes show a notable decrease in electrostatic potential on the wide groove of the G-quadruplex (Figure 6).

## DISCUSSION

We have examined the introduction of G·G·X·O tetrads into a human telomeric G-quadruplex. Unlike previously studied X·O·X·O tetrads (29,45), a G·G·X·O tetrad requires only two residues within the same G-tetrad to be modified, allowing a flexibility in the placement of O so as to avoid substitution into an *anti* position guanine. Considering this prerequisite, an X·O pair has been successfully integrated at five different positions on the *HT* G-quadruplex scaffold (based upon the *syn* position G-to-O substitution condition) without significantly affecting the structure or thermal stability. We also show that a G·G·X·O tetrad can be formed when O is substituted into an *anti* position guanine (*HT-21x17o*) while maintaining proper folding topology but at a cost of ∼12°C in thermal stability.

The complementary pairing of X and O in the modified G·G·X·O tetrad is demonstrated to be a versatile design for countering the destabilizing effects of single X or O modifications introduced on G-quadruplexes via DNA damage. Although the likelihood of a nearby occurrence of X and O mutations on a G-rich sequence of the genome is assumed to be small *in vivo*, such nearby mutations could favor one G-quadruplex form over other alternatives.

The X·O modification introduces a pH-depedent negatively charged groove on the molecule. Generation of a charged groove may affect G-quadruplex-protein interactions. From an engineering perspective, G-quadruplexes in therapeutic and sensing applications may have their binding properties enhanced by such a modification. Additionally, nano-scale wires built upon the G-quadruplex architecture may benefit from the introduction of negatively charged bases in a controlled manner.

## CONCLUSION

Single xanthine (X) or 8-oxoguanine (O) substitutions were found to be highly disruptive to the structure and stability of a G-quadruplex. In contrast, complementary X and O dual substitutions can lead to the formation of a G·G·X·O tetrad in a stable G-quadruplex. The solution structure of a G·G·X·O-containing G-quadruplex formed by a modified human telomeric sequence was solved, revealing the same folding topology in both the protonated and deprotonated forms. The X·O pair in the deprotonated state showed an enhanced electronegativity in the modified groove, with potential applications in charge-dependent recognition by other molecules.

## Supplementary Material

SUPPLEMENTARY DATA
