# Supplementary material for: Xanthine and 8-oxoguanine in G-quadruplexes: formation of a G·G·X·O tetrad
Source: Nucleic Acids Res. 2015 Sep 22;43(21):10506–14. doi: 10.1093/nar/gkv826 (PMC4666386; doi:10.1093/nar/gkv826)
Supplement: SUPPLEMENTARY DATA [file supp_43_21_10506__index.html]

Xanthine and 8-oxoguanine in G-quadruplexes: formation of a G·G·X·O tetrad — SUPPLEMENTARY DATA 

# Xanthine and 8-oxoguanine in G-quadruplexes: formation of a G·G·X·O tetrad

## SUPPLEMENTARY DATA

- SUPPLEMENTARY DATA
