# Supplementary material for: Xanthine and 8-oxoguanine in G-quadruplexes: formation of a G·G·X·O tetrad
Source: Nucleic Acids Res. 2015 Sep 22;43(21):10506–14. doi: 10.1093/nar/gkv826 (PMC4666386; doi:10.1093/nar/gkv826)
Supplement: SUPPLEMENTARY DATA [file supp_gkv826_SI.pdf]

Xanthine and 8-oxoguanine in G-quadruplexes: Formation of a G•G•X•O tetrad

Vee Vee Cheong, Brahim Heddi, Christopher Jacques Lech, and Anh Tuân Phan*

School of Physical and Mathematical Sciences, Nanyang Technological University, Singapore 637371

*To whom correspondence should be addressed. Tel: +65 6514 1915; Fax: +65 6795 7981;
Email: phantuan@ntu.edu.sg

SUPPORTING INFORMATION

Supplementary tables

Table S1. Single substituted sequences used this study and their thermal stability

Name	Sequence (5′ – 3′) *S A A S A A S S A S A A*	T_m (°C)	ΔT_m (°C)
HT-3x	TT **X**GG TTA GGG TTA GGG TTA GGG A	44.5 ± 0.1	-11.1
HT-4x	TT G**X**G TTA GGG TTA GGG TTA GGG A	30.3 ± 1.0	-25.4
HT-5x	TT GG**X** TTA GGG TTA GGG TTA GGG A	38.5 ± 0.3	-17.1
HT-9x	TT GGG TTA **X**GG TTA GGG TTA GGG A	36.7 ± 0.1	-18.9
HT-10x	TT GGG TTA G**X**G TTA GGG TTA GGG A	35.9 ± 0.8	-19.7
HT-11x	TT GGG TTA GG**X** TTA GGG TTA GGG A	39.3 ± 0.2	-16.3
HT-15x	TT GGG TTA GGG TTA **X**GG TTA GGG A	38.3 ± 0.8	-17.3
HT-16x	TT GGG TTA GGG TTA G**X**G TTA GGG A	32.7 ± 2.7	-22.9
HT-17x	TT GGG TTA GGG TTA GG**X** TTA GGG A	43.7 ± 0.5	-12.0
HT-21x	TT GGG TTA GGG TTA GGG TTA **X**GG A	38.5 ± 0.1	-17.1
HT-22x	TT GGG TTA GGG TTA GGG TTA G**X**G A	< 24.3*	-31.3
HT-23x	TT GGG TTA GGG TTA GGG TTA GG**X** A	36.8 ± 0.4	-18.8
HT-3i	TT **I**GG TTA GGG TTA GGG TTA GGG A	46.3 ± 0.8	-9.3
HT-4i	TT G**I**G TTA GGG TTA GGG TTA GGG A	43.1 ± 0.6	-12.5
HT-5i	TT GG**I** TTA GGG TTA GGG TTA GGG A	49.7 ± 0.6	-6.0
HT-9i	TT GGG TTA **I**GG TTA GGG TTA GGG A	45.3 ± 0.3	-10.3
HT-10i	TT GGG TTA G**I**G TTA GGG TTA GGG A	43.5 ± 0.5	-12.1
HT-11i	TT GGG TTA GG**I** TTA GGG TTA GGG A	50.4 ± 0.5	-5.2
HT-15i	TT GGG TTA GGG TTA **I**GG TTA GGG A	44.6 ± 0.5	-11.0
HT-16i	TT GGG TTA GGG TTA G**I**G TTA GGG A	47.1 ± 1.2	-8.5
HT-17i	TT GGG TTA GGG TTA GG**I** TTA GGG A	48.8 ± 0.7	-6.8
HT-21i	TT GGG TTA GGG TTA GGG TTA **I**GG A	43.7 ± 0.5	-11.9
HT-22i	TT GGG TTA GGG TTA GGG TTA G**I**G A	45.6 ± 0.4	-10.0
HT-23i	TT GGG TTA GGG TTA GGG TTA GG**I** A	33.9 ± 0.1	-21.7
HT-9o	TT GGG TTA **O**GG TTA GGG TTA GGG A	36.8 ± 0.4	-18.8
HT-16o	TT GGG TTA GGG TTA G**O**G TTA GGG A	< 24.3*	-31.3
HT-17o	TT GGG TTA GGG TTA GG**O** TTA GGG A	34.9 ± 0.5	-20.7

T_m values were determined in buffer containing 10 mM potassium phosphate, 10 mM potassium chloride, pH 7.0. The modified residues used are: **X** = xanthine, **O** = 8-oxoguanine, **I** = inosine.
The glycosidic conformation of residues in the G-tetrad core is in *syn* (*S*) or *anti* (*A*) orientation.
*T_m below the detection range in the UV melting experiment.

Table S2. ^{15}N-labeled *HT-17x9o* sequences used in this study for spectral assignment

Name	Sequence (5′ – 3′)								
HT-17x9o [3]	TT	[*G]GG	TTA	**O**GG	TTA	GG**X**	TTA	GGG	A
HT-17x9o [4]	TT	G[*G]G	TTA	**O**GG	TTA	GG**X**	TTA	GGG	A
HT-17x9o [5]	TT	GG[*G]	TTA	**O**GG	TTA	GG**X**	TTA	GGG	A
HT-17x9o [10]	TT	GGG	TTA	**O**[*G]G	TTA	GG**X**	TTA	GGG	A
HT-17x9o [11]	TT	GGG	TTA	**O**G[*G]	TTA	GG**X**	TTA	GGG	A
HT-17x9o [15]	TT	GGG	TTA	**O**GG	TTA	[*G]G**X**	TTA	GGG	A
HT-17x9o [16]	TT	GGG	TTA	**O**GG	TTA	G[*G]**X**	TTA	GGG	A
HT-17x9o [21]	TT	GGG	TTA	**O**GG	TTA	GG**X**	TTA	[*G]GG	A
HT-17x9o [22]	TT	GGG	TTA	**O**GG	TTA	GG**X**	TTA	G[*G]G	A
HT-17x9o [23]	TT	GGG	TTA	**O**GG	TTA	GG**X**	TTA	GG[*G]	A

X = xanthine, **O** = 8-oxoguanine, [*G] = ^{15}N-labeled guanine

Supplementary figures

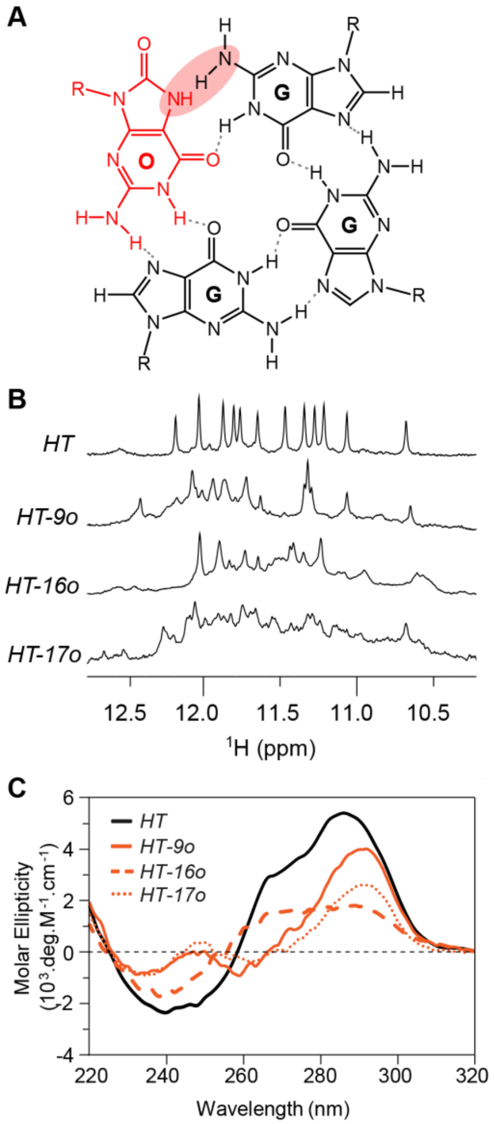

Figure S1. Characterization of *HT* and the modified constructs *HT-9o*, *HT-16o*, and *HT-17o*. (**A**) Arrangement of G-tetrad with a single substitution of 8-oxoguanine (O). Hydrogen-bond connectivity affected by the modification is shaded. (**B**) Imino proton spectra. (**C**) CD spectra.

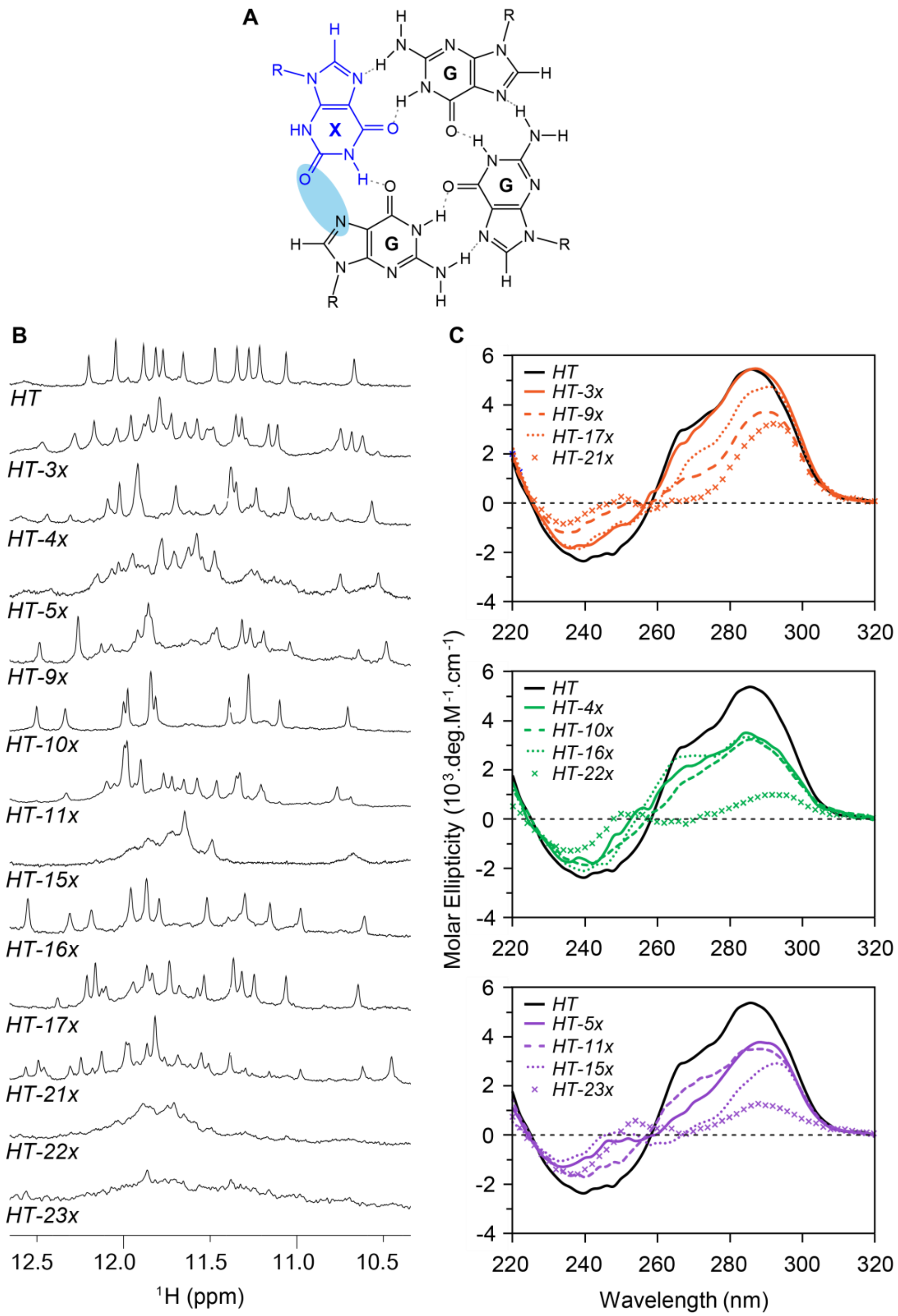

Figure S2. Characterization of *HT* and the constructs with single xanthine substitutions. (**A**) Arrangement of G-tetrad with a single substitution of xanthine (X). Hydrogen-bond connectivity affected by the modification is shaded. (**B**) Imino proton spectra. (**C**) CD spectra.

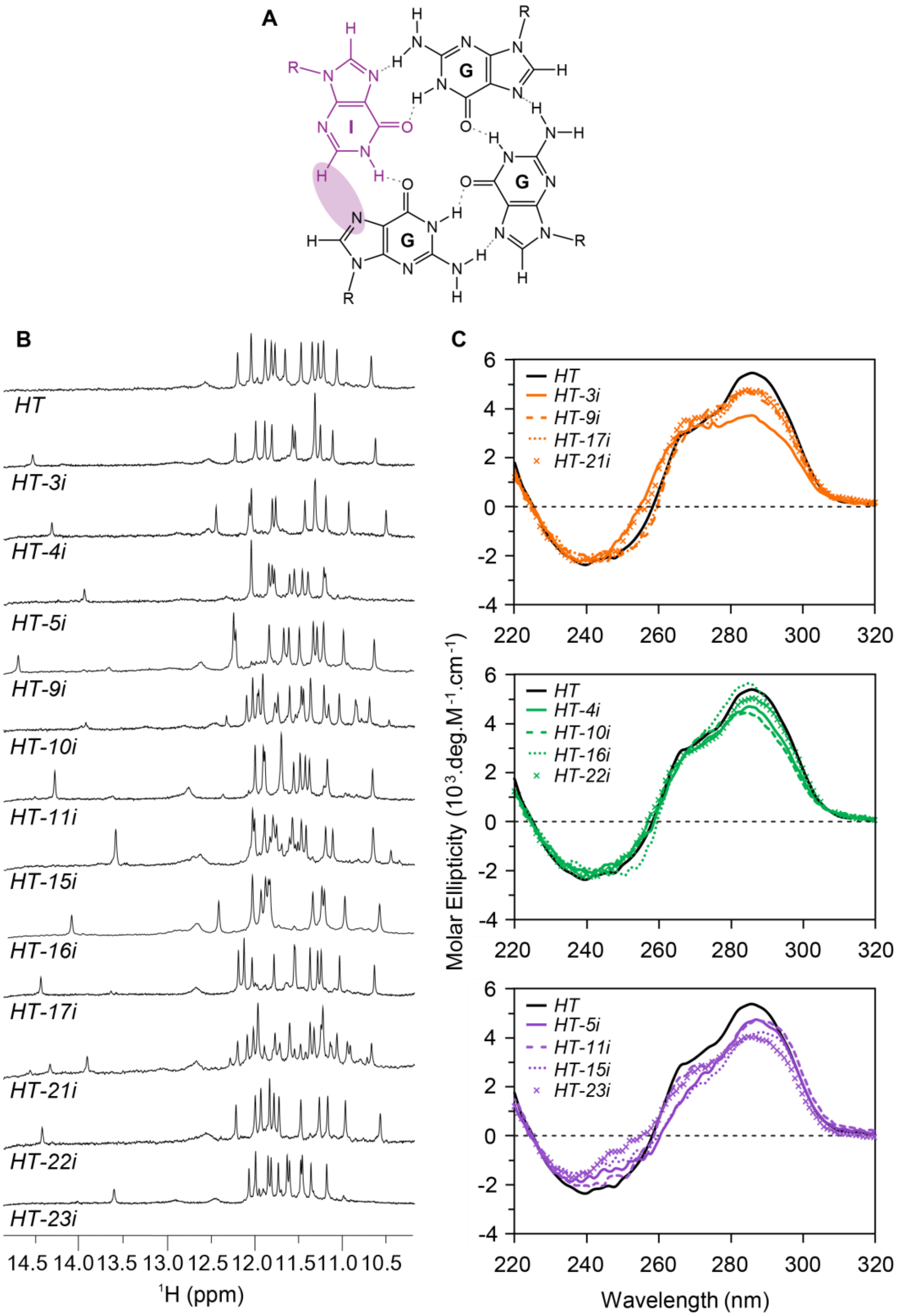

Figure S3. Characterization of *HT* and the constructs with single inosine substitutions. (**A**) Arrangement of G-tetrad with a single substitution of inosine (I). Hydrogen-bond connectivity affected by the modification is shaded. (**B**) Imino proton spectra. (**C**) CD spectra.

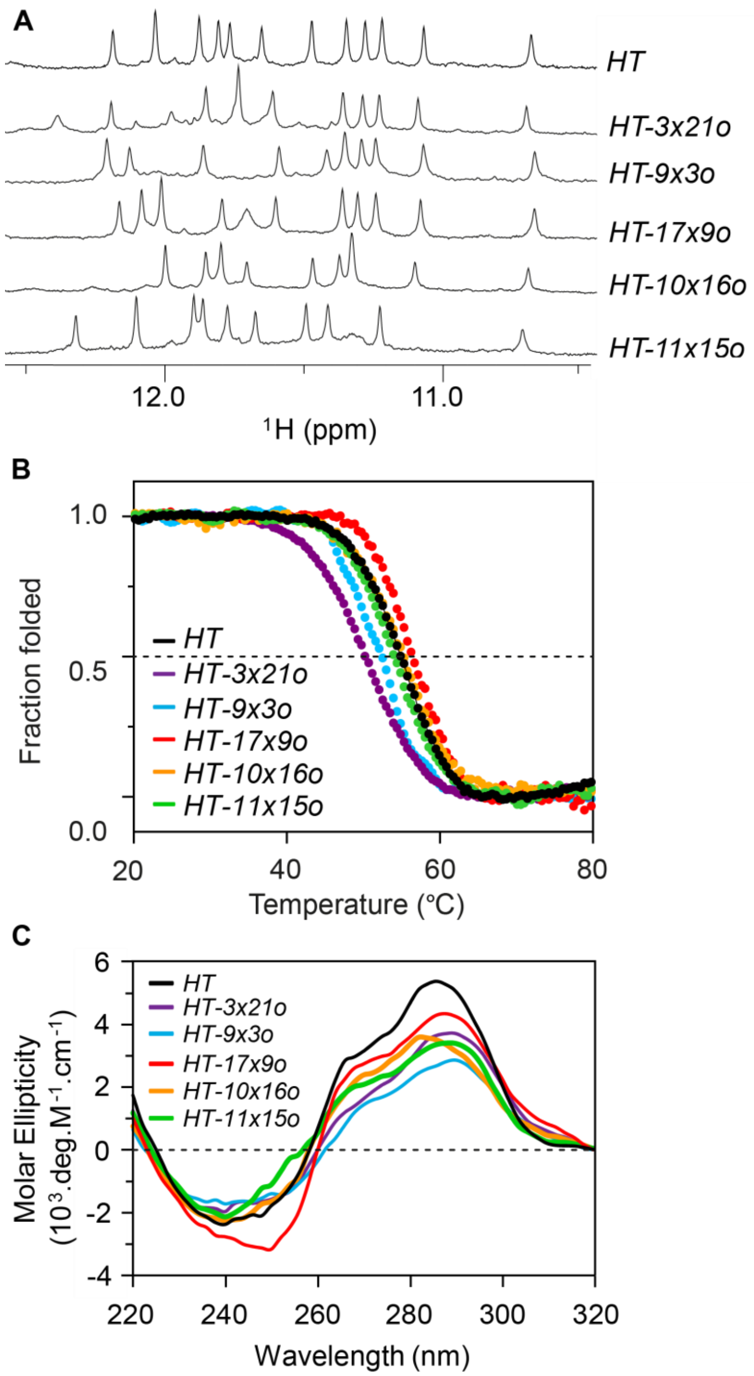

Figure S4. Characterization of *HT* and the constructs with dual X•O substitutions. (**A**) Imino proton spectra. (**B**) UV melting curves. (**C**) CD spectra.

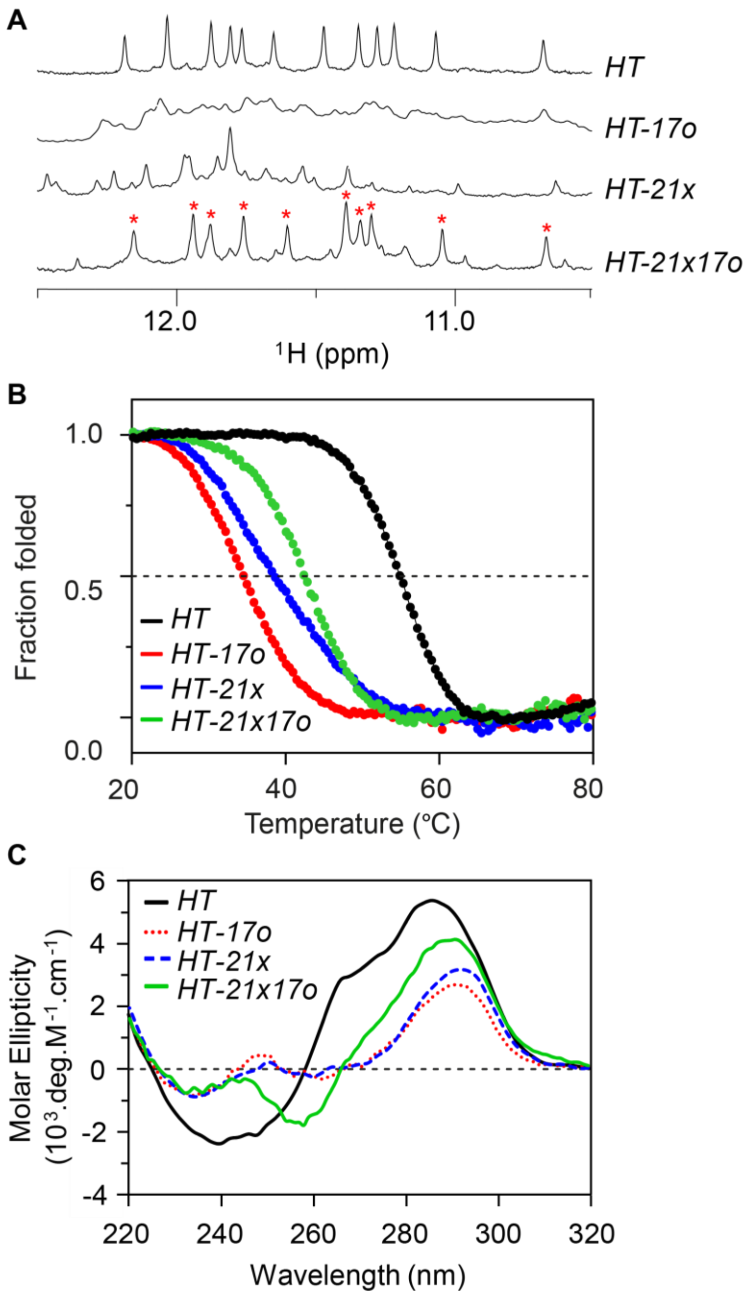

Figure S5. Characterization of *HT* and the constructs *HT-17o*, *HT-21x* and *HT-21x17o*. (**A**) Imino proton spectra. Imino peaks from the major form are indicated by red asterisks (*). (**B**) UV melting curves. (**C**) CD spectra.

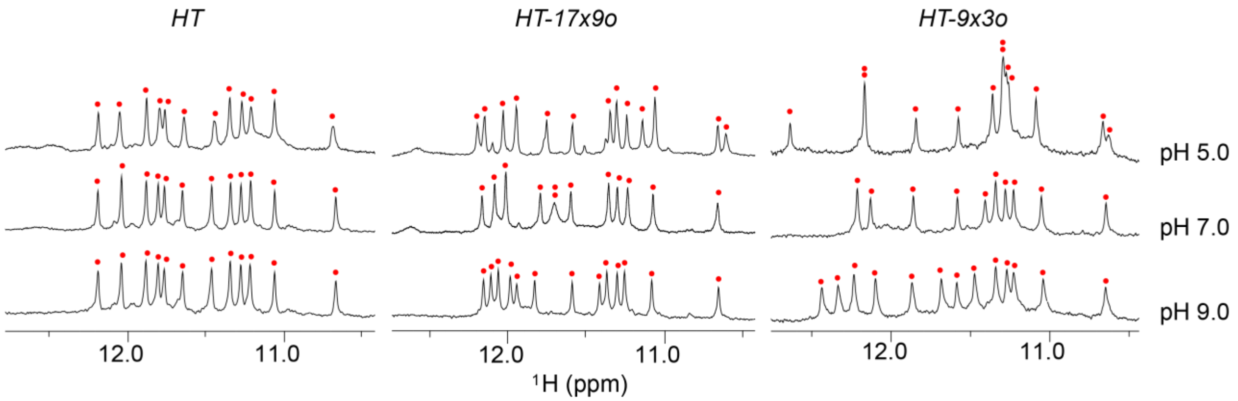

Figure S6. Imino proton spectra of *HT* (left), *HT-17x9o* (middle) and *HT-9x3o* (right) recorded at pH 5.0, 7.0 and 9.0. Each imino peak is indicated by a red dot above the spectrum.

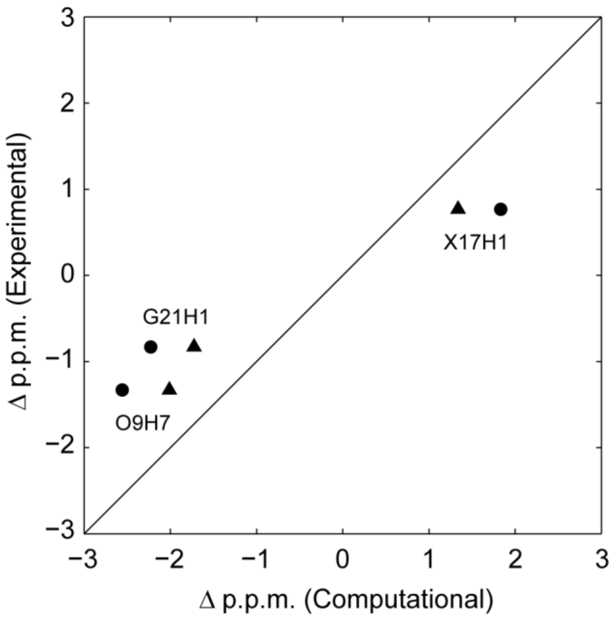

Figure S7. Comparison between experimental and computed values of pH-dependent NMR chemical shift variations of *HT-17x9o*. Experimental data are NMR chemical shifts observed for O9H7, X17H1 and G21H1 with change from the protonated (pH 5.0) to the deprotonated (pH 9.0) states. Computational data are computed changes in NMR chemical shifts of these protons in model G•G•X•O tetrads in the protonated and deprotonated states. Model tetrads were optimized at the HF/6-31G(d) level with NMR chemical shifts computed at the HF/6-31G(d) (▲) and MP2/6-31G(d) (●) level.

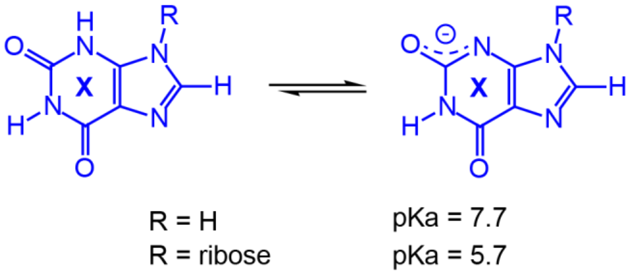

Figure S8. Acid dissociation constant (pKa) values of xanthine (X) which undergoes deprotonation at N3.

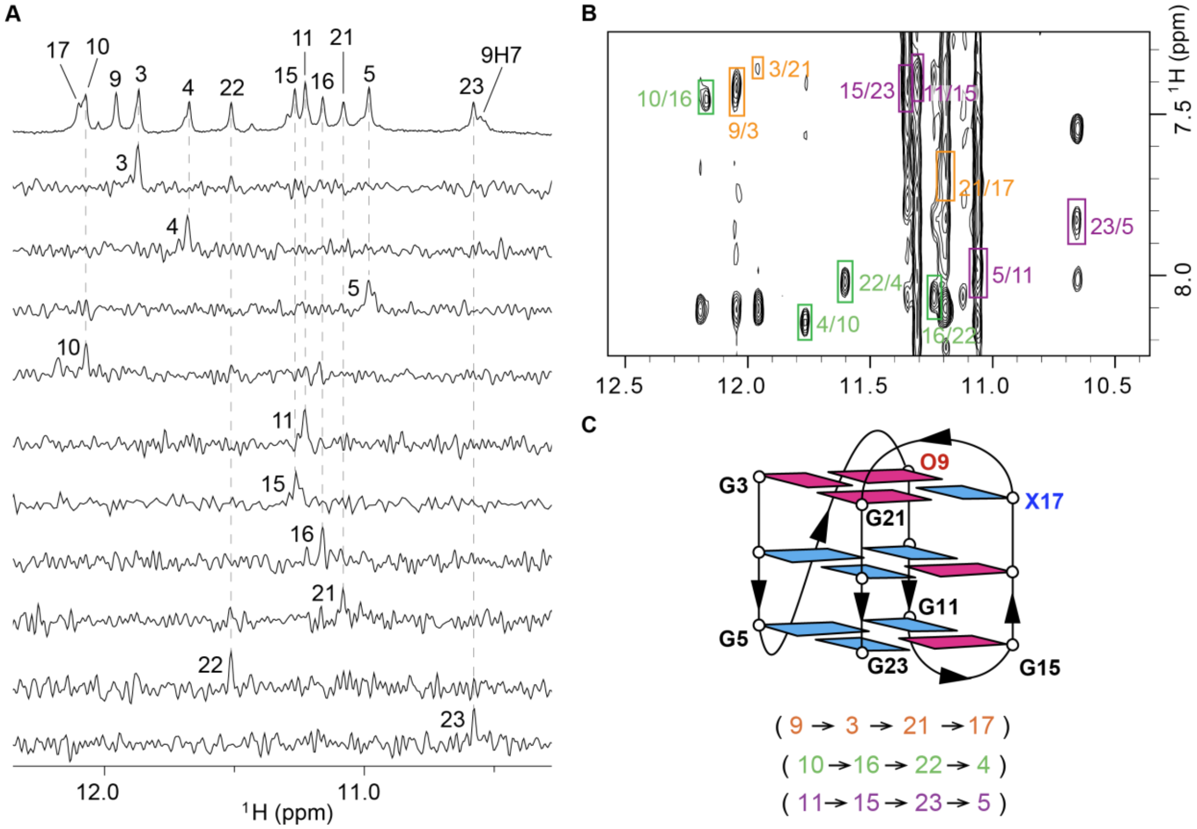

Figure S9. NMR proton spectra and assignments of *HT-17x9o* at pH 5.5. (**A**) ^{15}N-filtered spectra recorded on samples with 4%-^{15}N-enrichment at indicated positions. Assignment of O9H1, O9H7 and X17H1 were based on {^{1}H-^{15}N}-HSQC and through-space NOE correlation from NOESY spectrum. (**B**) NOESY spectrum of *HT-17x9o* (mixing time, 300 ms) used for the folding topology determination. H1-H8 cross-peaks are framed and labeled with the number of imino protons followed by that of H8. (**C**) Schematic representation of X•O-modified human telomeric G-quadruplex (*HT-17x9o*). *Syn* and *anti* guanines are colored in magenta and cyan respectively. Characteristic NOE connectivities for each G-tetrad layer of *HT-17x9o* are shown below, with top, middle and bottom G-tetrad colored as orange, green and purple respectively.

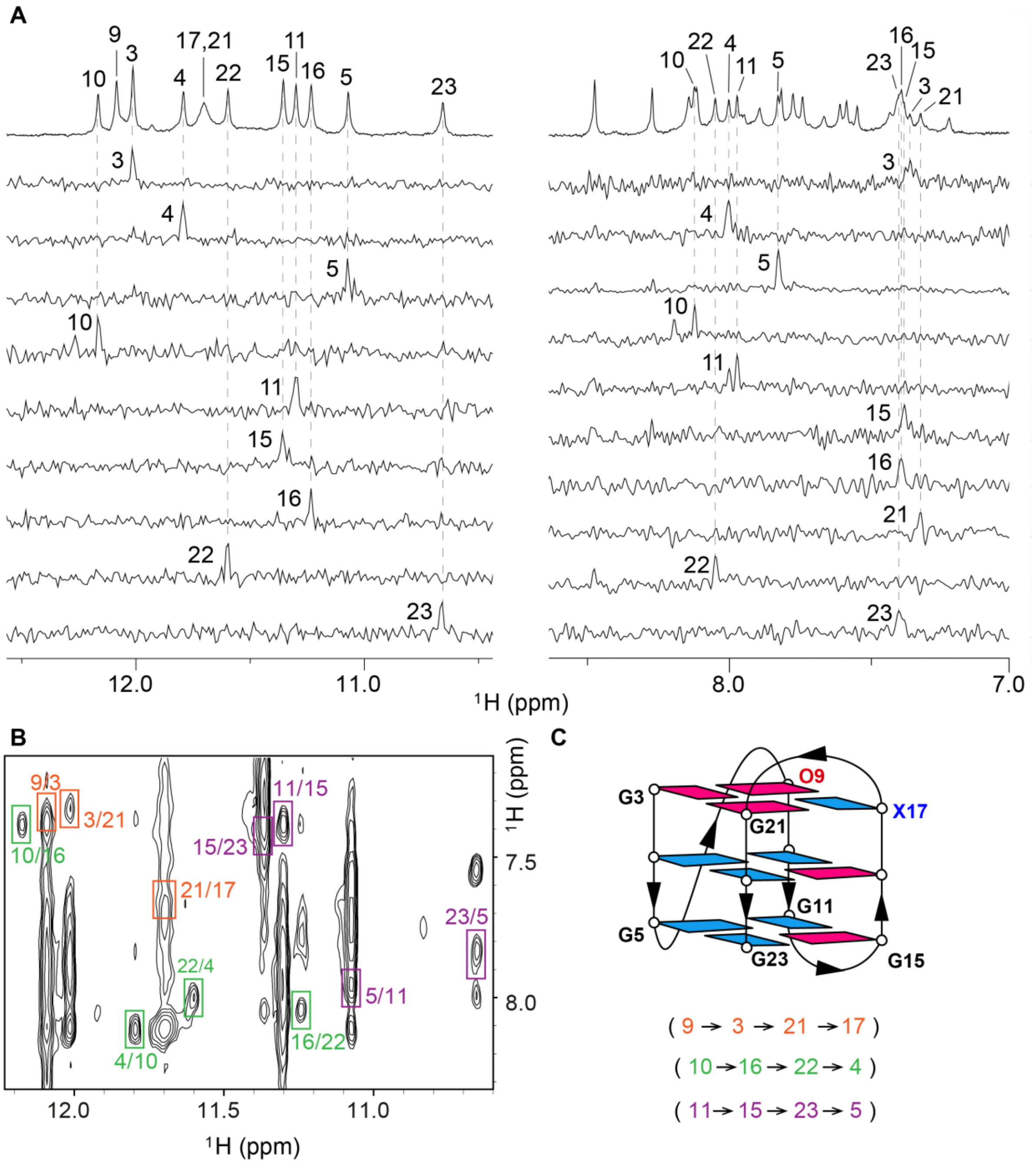

Figure S10. NMR proton spectra and assignments of *HT-17x9o* at pH 7.0. (**A**) ^{15}N-filtered spectra of the imino proton region (left) and aromatic proton region (right) recorded on samples with 4%-^{15}N-enrichment at indicated positions. (**B**) NOESY spectrum of *HT-17x9o* (mixing time, 300 ms) used for the folding topology determination. H1-H8 cross-peaks are framed and labeled with the number of imino protons followed by that of H8. (**C**) Schematic representation of X•O-modified human telomeric G-quadruplex (*HT-17x9o*). *Syn* and *anti* guanines are indicated in magenta and cyan respectively. Characteristic NOE connectivities for each G-tetrad layer of *HT-17x9o* are shown below, with top, middle and bottom G-tetrad colored as orange, green and purple respectively.

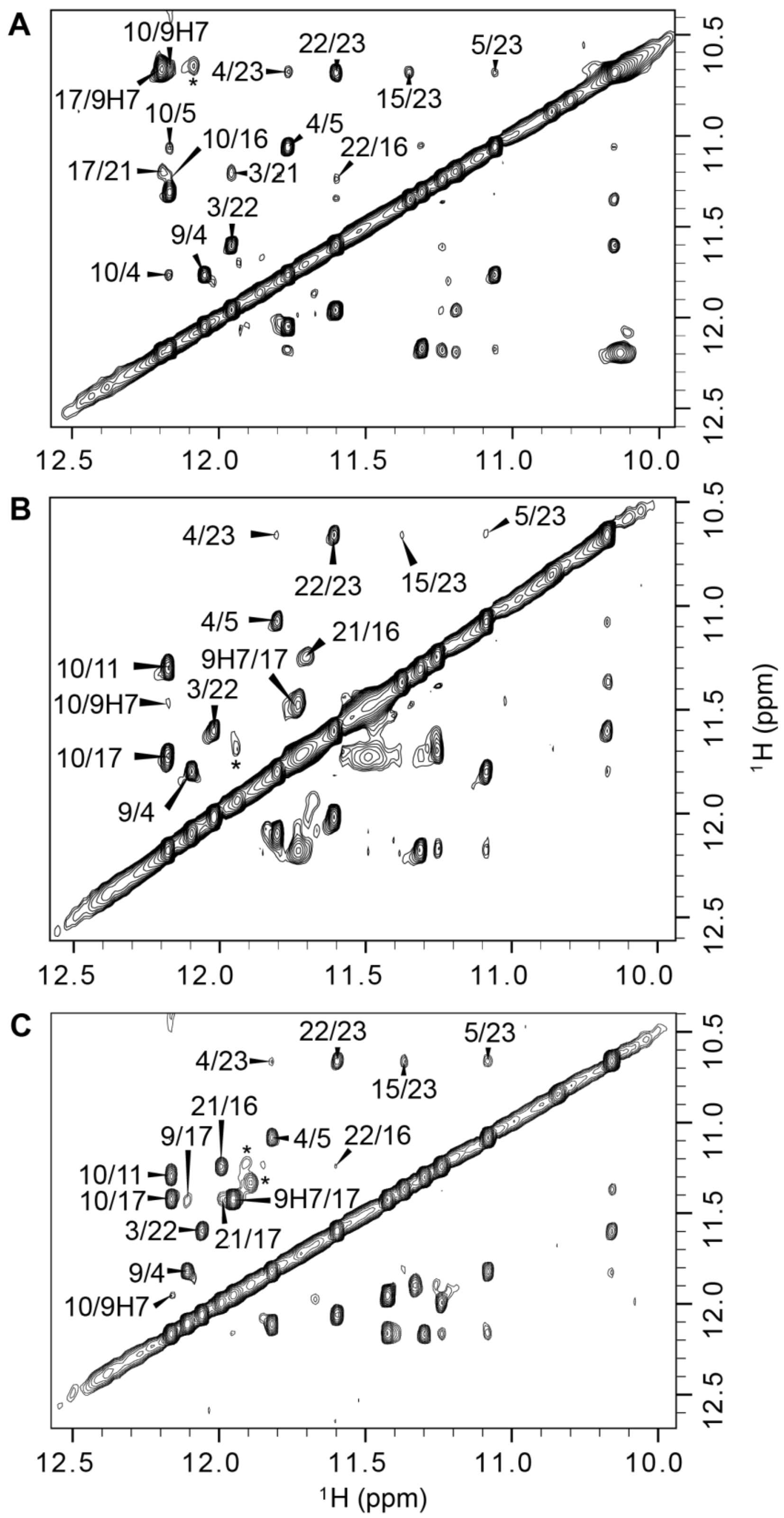

Figure S11. NOESY spectrum of *HT-17x9o* recorded at (**A**) pH 5.5, (**B**) pH 7.0 and (**C**) pH 8.0, with H1-H1/H7-H1/H1-H7 through space correlations labeled as indicated on the spectrum. NOE correlations from minor form are marked by asterisks (*).

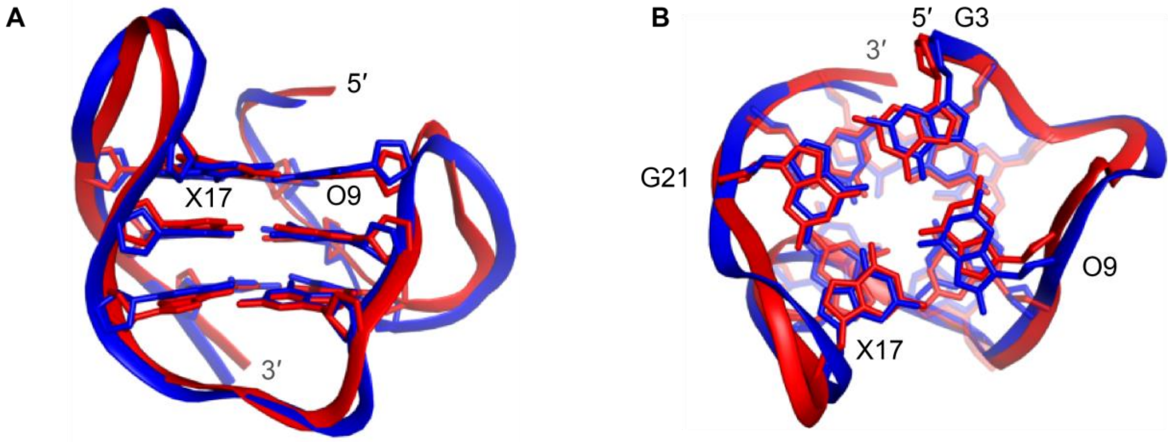

Figure S12. Comparison between *HT-17x9o* (colored in blue) and *HT* G-quadruplex (colored in red). Structures were aligned based on their G-tetrad core with RMSD 0.649 Å. (**A**) Side view and (**B**) top view of the superimposed structures, with the sugar phosphate backbone and bases of G-tetrad core presented. Residues 1, 2, 18 and 19 are removed in the top view for clarity.
